# Timing of Meals and Sleep in the Mediterranean Population: The Effect of Taste, Genetics, Environmental Determinants, and Interactions on Obesity Phenotypes

**DOI:** 10.3390/nu15030708

**Published:** 2023-01-30

**Authors:** Rocío Barragán, Rebeca Fernández-Carrión, Eva María Asensio-Márquez, Carolina Ortega-Azorín, Andrea Álvarez-Sala, Alejandro Pérez-Fidalgo, José Vicente Sorlí, Olga Portolés, Inmaculada González-Monje, Marie Pierre St-Onge, Dolores Corella

**Affiliations:** 1 Department of Preventive Medicine and Public Health, School of Medicine, University of Valencia, 46010 Valencia, Spain; 2 CIBER Fisiopatología de la Obesidad y Nutrición, Instituto de Salud Carlos III, 28029 Madrid, Spain; 3 Center of Excellence for Sleep & Circadian Research, Department of Medicine, Columbia University Irving Medical Center, New York, NY 10032, USA; 4 Department of Medical Oncology, Hospital Clínico Universitario Valencia, 46010 Valencia, Spain; 5 CIBERONC, Instituto de Salud Carlos III, 28029 Madrid, Spain; 6 Division of General Medicine, Department of Medicine, Columbia University Irving Medical Center, New York, NY 10032, USA

**Keywords:** sleep, mealtimes, taste perception, obesity, Mediterranean diet

## Abstract

Circadian rhythms regulate the sleep–wake and feeding–fasting cycles. Sleep and feeding constitute a complex cycle that is determined by several factors. Despite the importance of sleep duration and mealtimes for many obesity phenotypes, most studies on dietary patterns have not investigated the contribution of these variables to the phenotypes analyzed. Likewise, they have not investigated the factors related to sleep or mealtimes. Thus, our aims were to investigate the link between taste perception and eating/sleep patterns and to analyze the effect of the interactions between sleep/meal patterns and genetic factors on obesity phenotypes. We conducted a cross-sectional analysis on 412 adults from the Mediterranean population. We measured taste perception (bitter, sweet, salty, sour, and umami) and assessed sleep duration and waketime. The midpoint of sleep and social jetlag was computed. From the self-reported timing of meals, we estimated the eating window, eating midpoint, and eating jetlag. Adherence to the Mediterranean diet was measured with a validated score. Selected polymorphisms in the TAS2R38, CLOCK, and FTO genes were determined, and their associations and interactions with relevant phenotypes were analyzed. We found various associations between temporal eating, sleep patterns, and taste perception. A higher bitter taste perception was associated with an earlier eating midpoint (*p* = 0.001), breakfast time (*p* = 0.043), dinner time (*p* = 0.009), waketime (*p* < 0.001), and midpoint of sleep (*p* = 0.009). Similar results were observed for the bitter taste polymorphism TAS2R38-rs713598, a genetic instrumental variable for bitter perception, increasing the causality of the associations. Moreover, significant gene–sleep interactions were detected between the midpoint of sleep and the TAS2R38-rs713598 (*p* = 0.032), FTO-rs9939609 (*p* = 0.037), and CLOCK-rs4580704 (*p* = 0.004) polymorphisms which played a role in determining obesity phenotypes. In conclusion, our study provided more information on the sleep and mealtime patterns of the general Spanish Mediterranean population than on their main relationships. Moreover, we were able to show significant associations between taste perception, specifically bitter taste; sleep time; and mealtimes as well as an interaction between sleep time and several genetic variants linked to obesity phenotypes. However, additional research is needed to better characterize the causality and mechanisms behind these associations.

## 1. Introduction

Numerous physiological, metabolic, and behavioral systems, including the sleep–wake and feeding–fasting cycles, are regulated by circadian rhythms [1,2]. The molecular oscillators responsible for these functions are known as circadian clocks [3]. These include the hypothalamus suprachiasmatic nucleus, which contains the brain’s primary clock, and different clocks in the peripheral tissues that orchestrate 24 h cycles at the transcriptional level [4,5]. Several core clock genes are involved in the control of this system, and the underlying mechanism has already been described [6]. Among the most significant is the CLOCK (circadian locomotor output cycles kaput) gene. This core clock gene variation has been linked to obesity [7,8], diabetes, and other related phenotypes [9,10].

In recent decades, interest in the fundamental behaviors of sleeping and eating has increased. The prevalence of sleep disorders and inadequate sleep has risen worldwide [11,12]. This fact is closely linked to an increased risk of obesity [13], coronary heart disease, stroke, and hypertension [14]. Some authors have indicated that the increased risk of disease may be due to a combination of factors, nutrition most likely being the key component in this association [14,15,16,17]. Notably, the feeding cycle and, more specifically, eating patterns, including all food-related behaviors regardless of intake, such as eating frequency, meal timing, and eating window [18], have been independently associated with cardiometabolic diseases [19,20,21,22], including obesity [23,24]). Eating is a complex pattern that varies between cultures, social groups, and individuals, leading to variations in what we eat and when we eat it [23]. Diverse factors have an impact on food selection and preferences [25,26], the ability to perceive distinct tastes being the most prominent one [27]. Taste is one of the most important factors in food choice, as people prefer to consume foods that they enjoy [28,29,30]. Variability in taste perception is not well understood, but we have demonstrated in earlier papers that age decreases the intensity of taste perception, that women score higher on these tests, and that some single-nucleotide polymorphisms (SNPs) may influence taste perception [31,32]. To date, only polymorphisms in the TAS2R38 (taste 2 receptor member 38) gene have been consistently associated with variations in bitter taste perception in different populations [33,34], including the Mediterranean population [31]. Specifically, the rs713598 polymorphism (Ala49Pro amino acid change) in this gene has been the most widely analyzed as a tag SNP [31,34]. Polymorphisms in the FTO (fat-mass- and obesity-associated) gene, a well-known gene strongly associated with obesity phenotypes in genome-wide association studies (GWASs) [35], have also been associated with the perception and intake of sweet substances [36]. Hence, the FTO-rs11642841 SNP has been related to sugar intake, and the rs9939609 SNP, highly correlated within the FTO gene, has been associated with sweet taste perception [37]. 

Despite the notion that taste perception is a crucial aspect of food consumption, its association with obesity remains controversial [32,38,39,40,41,42]. Moreover, taste perception has been largely ignored in research related to dietary patterns. In relation to sleep patterns, however, researchers have examined the relationship between taste perception and sleep duration with mixed results. Martelli et al. [43] conducted an observational study which revealed that the total sleep time was only positively correlated with sweet taste perception. Nonetheless, in an intervention sleep restriction study, a reduction in sleep duration (−2 h of habitual sleep) did not affect the intensity of sweet taste perception, but a greater preference for sweet foods was observed [44]. Similar results in relation to food preferences were found in other investigations [45,46]. Lv et al. also observed an increase in the strength of umami and sour taste perception in those who rated their sleepiness as intense [46]. In a group of 11,030 Chinese people, a short sleep duration, insomnia, and sleepiness were associated with impaired taste perception as determined by a questionnaire [47]. Nevertheless, the temporality of the associations remains to be determined.

Thus, there is a need to incorporate taste-related phenotypic and genetic factors into studies examining the relationship between sleep patterns and taste perception as well as to extend the analysis of these associations to the timing of food intake when considering eating patterns. In addition, eating and sleep patterns may be important determinants of obesity phenotypes, mainly through gene–lifestyle interactions [48]. Therefore, we hypothesized that taste perception, a complex variable related to food intake; satiation; and a number of phenotypes may have a significant association with eating and sleep patterns via diverse mechanisms. Due to the association between sleep and/or meal patterns and obesity, we thought that sleep and eating patterns may interact with obesity polymorphisms, thereby modulating the effect of these genetic factors on obesity phenotypes. The current study sought to determine whether (1) there is a link between taste perception (bitter, sweet, salty, sour, and umami) and chronobiological sleep and eating patterns, (2) there is a link between the selected relevant genetic variants (TAS2R38, FTO, and CLOCK polymorphisms) and sleep and eating patterns, and (3) the relationship between the selected SNPs and obesity phenotypes is modulated by sleep and eating patterns in the Mediterranean population.

## 2. Materials and Methods

### 2.1. Study Design and Participants

We conducted a cross-sectional analysis on a subsample of participants in the Obesity, Nutrition & Information and Communication Technologies (OBENUTIC) study carried out in the Mediterranean population. The OBENUTIC study has previously been described in detail [31]. Briefly, it is an open case-control study on obesity in Caucasian adults recruited from the general population of the Mediterranean region of Valencia, Spain, with the main objective of examining diverse environmental and genetic factors associated with obesity phenotypes. This was a general population cohort enriched with obesity cases (BMI ≥ 30 kg/m^2^) in comparison with nonobese controls. Controls were unpaired by sex and age in obesity cases to allow more general cross-sectional analyses from the same geographical area. Both obesity cases and controls contained apparently healthy individuals recruited through advertisements in shopping malls; cultural associations; and other types of places in the general area, such as public and private institutions, educational centers, and other contacts. The exclusion criteria were being pregnant or breast-feeding; suffering from some type of infectious/contagious disease; having invalidating physical or psychological diseases, such as cancer diagnosis, thyroid alterations, or Cushing disease; having conditions that could alter gustatory functions (e.g., tooth extraction), high alcohol intake; or the consumption of other drugs. We included men and women between the ages of 18 and 80 years old and obtained their sociodemographic, lifestyle, clinical, biochemical, anthropometric, and taste perception data as well as stored biological samples for further genomics and other omics determinations. Likewise, the collection of lifestyle data was extended for specific projects. Initially, the OBENUTIC cohort began in 2007 and only included the bitter taste perception tests, but, later, the project was completed by including perception tests for the other tastes. Likewise, information on sleeping and eating patterns was assessed later. In this study, we included 419 participants (men and women) who consecutively had the complete data available for the perception tests of bitter, sweet, sour, salty, and umami tastes as well as information on the sleep variables and genetic data for the selected polymorphisms in addition to the general variables. These subjects completed the study between 2015 and 2017. No influence of the coronavirus pandemic was present on the assessed variables. Additionally, being shift worker was an additional exclusion criterion for final data analysis after taking into account the influence of this condition on sleep patterns. Thus, from the preselected sample of 419 subjects, 7 participants were excluded from the final analysis, as they were shift workers, leaving a final sample of 412 participants. Timing of food intake and other eating patterns were only available for 73.3 % of those individuals, resulting in a sample size of 302 participants for the analysis including timing of meal variables. Participants provided written informed consent, and the protocol and methods were approved by the Human Research Ethics Committee of the University of Valencia, Valencia (reference numbers: R1326, 21/03/2006, and H1488282121722, 06/04/2017).

### 2.2. Demographic, Anthropometric, Biochemical, Clinical, and Lifestyle Variables

The sociodemographic, lifestyle, medication use, and clinical variables were obtained through a standardized questionnaire previously used in our studies [31]. For lifestyle variables, diet pattern was evaluated using the validated 14-item scale to assess adherence to the Mediterranean diet (MedDiet) as previously reported [49]. Low physical activity was determined if the participant walked less than 20 min a day, and, for tobacco smoking, subjects were classified as current or noncurrent smokers according to their self-reported smoking habits. Participants’ weights and heights were measured directly with calibrated scales (TANITA-BC-420-S, Tanita UK Ltd., Middlesex, UK) and a stadiometer (SECA Mod 220, Seca Deutschland Gmbh & Co. Kg., Hamburg, Germany), respectively, by trained personnel and according to standard recommendations. BMI was calculated as weight in kilograms divided by height in square meters. Waist circumference was measured halfway between the lowest rib and the iliac crest after normal exhalation using an anthropometric tape. Blood pressure was measured twice using a validated semiautomatic oscillometer (Omron HEM-705CP, OMRON Healthcare Europe B.V., Hoofddorp, Netherlands). 

Blood samples were collected after a 12 h overnight fast. In a certified clinical laboratory, plasma concentrations of fasting glucose, total cholesterol, HDL-cholesterol, and triglycerides were measured using enzymatic methods as previously described [31], and LDL-C was estimated using the Friedewald equation (Olympus AU5400, Beckman Coulter, CA, USA). Diabetes was defined as having a fasting glucose level of 126 mg/dL or taking diabetes medication.

### 2.3. Taste Perception Tests

Taste perception tests (for bitter, sweet, salty, sour, and umami) were carried out in the Genetic and Molecular Epidemiology Unit in the sensory research laboratory, Department of Preventive Medicine and Public Health, University of Valencia, Valencia, by trained personnel using the same methodology, which was standardized for that purpose, as previously reported [31,32]. Briefly, the test was undertaken in the morning under standard conditions with comfort and silence to allow the participants to be focused on the test. Trained staff provided a detailed explanation of the procedures prior to starting the series of tests. Participants were exposed to five concentrations for each of the five tastes (bitter, sweet, salty, sour, and umami) using a standard tastant for each taste: phenylthiocarbamide (PTC), sucrose, NaCl, citric acid, and L-glutamic acid monopotassium salt monohydrate (MPG), respectively (Sigma-Aldrich, Milan, Italy). Distilled water was used as the solvent. Five solutions of different concentrations (I to V) were prepared for each tastant, additionally including a distilled-water control. The series of concentrations (I to V) used for each tastant has already been reported in detail [31,32]. Bitter taste perception tests were undertaken on strips of filter paper as previously reported [31,32]. The other tastants were prepared and tested in liquid form, dissolved to the concentration indicated, and were presented in different small colored tubes for each taste, labelled with symbols and organized into racks of a preset order, and the same preparation was performed for all participants. Before beginning the taste perception tests, participants had to rinse their mouths several times with water. All participants were given a template on which they had to complete a scale of taste perception intensity ratings for each taste and concentration. Subjects assessed the intensity of tastants on an ordinal scale consisting of 6 intensity values from 0 (no taste) to 5 (extremely strong) for all the tastes. Although, in our study, the participants rated 5 concentrations of each tastant, these data were used to validate the tests in a dosage–response manner as previously reported [31,32]. Here, for the present association study, the statistical analyses were only conducted with the rating scored for the maximum concentration of each series (concentration V), which was best for maximizing the differences between individuals. Therefore, for each tastant, the concentrations tested were as follows: 5.6 mM for PTC, 400 mM for sucrose, 200 mM for NaCl, 34 mM for citric acid, and 200 mM for MPG. Using the scores for the five taste qualities, we also classified the participant as taster when the score was ≥3 points or as nontaster when the score was less than 3 points.

### 2.4. Temporal Eating Patterns 

Temporal eating patterns represent all the modifiable behaviors regardless of intake [18]. Information about eating patterns was obtained from 302 participants using a standardized questionnaire that included specific questions. Participants were asked about their mealtimes on weekends (Saturday and Sunday, separately) and on weekdays (average of Monday through Friday). They were specifically asked about their breakfast; “almuerzo”, or mid-morning snack in this Mediterranean population; lunch; “merienda”, or afternoon tea in this Mediterranean population; and dinner schedules. This region’s traditional meal, “almuerzo,” occurs between breakfast and lunch. In Spain, “merienda” is the term for the meal that occurs between lunch and dinner. From these data, we computed the so-called eating window, the eating midpoint, and the eating jetlag as follows: Eating window (h) = [((5 × (dinner time − breakfast time on weekdays)) + (dinner time − breakfast time on Saturday) + (dinner time − breakfast time on Sunday)]/7; eating midpoint was calculated as the mean value between breakfast and dinner time over the entire week. The formula used for the eating midpoint week average was the same as that for eating window; and eating jetlag was calculated as the difference in midpoint of the eating period between workdays and weekend [2].

### 2.5. Sleep Patterns 

We gathered information from the 412 participants on weekday and weekend sleep-related items. Participants were asked: (1) How many hours do you sleep on weekdays? (2) How many hours do you sleep on Saturdays? (3) How many hours do you sleep on Sundays? In addition, we asked the same questions, but, this time, related them to their time of awakening. From these data, we calculated the following variables: average sleep duration (h) = ((5 × sleep duration on weekdays) + sleep duration on Saturdays + sleep duration on Sunday)/7; average waketime = ((5 × awakening time on weekdays) + awakening time on Saturdays + awakening time on Sunday)/7. Midpoint of sleep was obtained from the sleep duration and the time of awakening: midpoint of sleep (h) = waketime − (sleep duration/2). Social jet lag was calculated as the absolute value of the discrepancy between midpoint of sleep on workdays and the midpoint of sleep on weekends [50].

### 2.6. DNA Isolation and DNA Genotyping

Genomic DNA was isolated from white blood cells as previously reported [31]. The TAS2R38-rs713598, FTO-rs9939609, and CLOCK-rs4580704 polymorphisms were genotyped using an ABI Prism 7900HT Sequence Detection System (Applied Biosystems, Foster City, CA, USA) and using a TaqMan fluorescent allelic discrimination assay. For quality control purposes, 10% of randomly selected samples were genotyped a second time, and there were no discrepancies. All polymorphisms were in Hardy–Weinberg equilibrium (*p* = 0.16 for TAS2R38-rs713598, *p* = 0.06 for FTTO-rs9939609, and *p* = 0.97 for CLOCK-rs4580704). Additive and dominant models were tested for associations.

### 2.7. Statistical Analysis

Analyses were performed on the entire sample studied and were stratified by sex for the descriptive analysis. Chi-square tests were used to compare proportions. Student’s t-tests and ANOVA tests were applied to compare raw means of continuous variables. Normal distribution for continuous variables was tested using frequency-plotted histograms to quickly visualize the distribution of the single variables of interest. For skewed distribution, we used mathematical functions (logarithmic or square root functions) to transform the data to normal distributions. Thus, triglyceride concentrations were logarithmically transformed, and the eating jet lag and the social jet lag were square root transformed for statistical analyses. Anthropometric, blood pressure, biochemical, taste perception measurement, eating pattern, and sleep pattern data were expressed as mean ± SD (standard deviation). Genotypic variables were initially used as three categories, and then the additive and dominant models were tested using the model that best matched the data for each polymorphism. ANOVA, multivariable-adjusted, and covariance models were used to test the association between taste perception, polymorphisms, and eating/sleep patterns (all considered as continuous dependent variables). The models were sequentially adjusted as follows: Model 1 was unadjusted; Model 2 was adjusted for age and sex; and Model 3 was additionally adjusted for age, sex, obesity (when appropriate), adherence to MedDiet, smoking, and medications (lipid-lowering, antidiabetic, and antihypertensive drugs). An additional adjustment was made for low physical activity due to its association with obesity phenotypes (BMI and waist circumference). To study the role of gene–pattern interactions in determining BMI and waist circumference, we used multivariate linear models including main effects and interaction terms. Interactions were depicted by dichotomizing the sleep pattern variables and by using the dominant or recessive models for polymorphisms. Initial sample size calculations were carried out taking into account the eating pattern variables, as this information was available in a small number of subjects. We selected the timing of breakfast as the dependent variable and computed the number of subjects needed to obtain a correlation coefficient of at least r = 0.17 with the measured taste variable having a standard 80% of power for detecting associations (at an alpha error of 5% and a beta error of 20%). According to this estimation and using the corresponding formula, the minimum sample size required was 269 individuals. Our current sample size (302 or 412, depending on the outcome) was powerful enough to detect such associations and even small correlations. No correction for multiple comparisons was computed. Statistical analyses were performed with IBM SPSS Statistics version 28.0 for Windows (IBM Corp., Armonk, NY, USA). All tests were two-tailed, and *p* values < 0.05 were considered statistically significant for these associations.

## 3. Results

### 3.1. General Characteristics of the Population

The demographic, clinical, lifestyle, and taste perception test results are presented in Table 1. Of the 412 participants, 65.8% were female (mean age of 46.5 ± 13.9 years), and 30.9% were obese. A sex-specific analysis showed that males had a higher prevalence of obesity compared to females (*p* = 0.038) and that females perceived all the tastes except umami more intensely than males (*p* < 0.001 for bitter, *p* = 0.005 for sweet, *p* = 0.009 for salty, and *p* < 0.001 for sour).

Regarding sleep patterns, mean sleep duration was 7 h and 48 min with a waketime of 7:10 ± 0:53 h. No statistically significant sex-specific differences in sleep patterns were detected per sex (*p* < 0.05) (Table 2).

The data on eating patterns were available for 302 participants (73.3%). The timing of lunch was 14:39 ± 0:35 h, with females eating lunch later than males (*p* = 0.041). The average timing for the mid-morning snack meal was 10:53 ± 0:33 h, which was, again, later for females than for males (*p* < 0.001) (Table 3).

### 3.2. Association between Phenotypic (Measured) Taste Perception, Candidate SNP, and Eating Patterns

Table 4 shows the association between taste perception and eating patterns, including the timing of meals and adherence to the Mediterranean diet pattern. Participants with a higher score for the bitter taste test had an earlier timing of breakfast, timing of dinner, and eating midpoint (*p* < 0.05) after adjustments for sex and age. For sweet perception, those with a higher perception of the sweet taste had a later eating midpoint (*p* = 0.030), a later breakfast time (*p* = 0.002), and a greater eating jetlag (*p* = 0.031). Moreover, a higher perception of the sweet taste was associated with a higher adherence to the MedDiet (*p* = 0.022). A higher score in the sour (*p* = 0.048) and umami (*p* = 0.031) perception tests was related to a later breakfast time. No significant associations were detected between salt perception and eating patterns. Adjustments for sex, age, obesity, smoking, and medication did not change these results apart from the results obtained for the sweet taste with respect to the eating midpoint (*p* = 0.050), eating jetlag (*p* = 0.079), umami taste, and breakfast time (*p* = 0.070). In Table 4, the eating pattern variables were computed by combining the information corresponding to the weekdays and the weekend, as this combined variable represents the average for the pattern. However, additional information corresponding to the separate variables for the weekdays and weekend had statistically significant associations with the measured taste variables is presented in Table S1.

Table 5 shows the associations between the candidate genes and eating patterns. We selected three genes as detailed in the methods (the TAS2R38 gene, related to bitter taste perception; FTO, the obesity-associated gene; and the CLOCK gene, one of the core clock genes involved in the regulation of the circadian timing system). See Table S2 for genotype prevalence. Consistent with the taste perception rating, TAS2R38-rs713598 was also related to the eating midpoint and breakfast time, the CCs, or tasters, being those who had an earlier breakfast time (*p* = 0.029) and eating midpoint (*p* = 0.008). Being carriers of the obesity risk allele (AA) in FTO-rs9939609 was associated with an earlier lunch (*p* = 0.021) and afternoon teatime (*p* = 0.038) in the Mediterranean population. However, for the clock polymorphism (rs4580704), we did not find any significant association. Findings remained statistically significant even after additional adjustments for obesity, smoking, and medication. Furthermore, a new significant association was noticed between FTO-rs9939609 (AA carriers) and a shorter eating window (*p* = 0.047) following adjustments.

Although adherence to the MedDiet was classified as an eating pattern, we also evaluated the association of this Mediterranean diet pattern with eating and sleep characteristics. A higher adherence to the MedDiet was related to a later afternoon teatime meal (β = 0.05 ± 0.02; *p* = 0.043) and a longer sleep duration (β = 0.07 ± 0.03; *p* = 0.010) (Figure S1).

In Table 5, the eating pattern variables were computed by combining the information corresponding to the weekdays and the weekend, as this combined variable represents an average for the pattern. However, additional information corresponding to the separate variables for the weekdays and weekend had statistically significant associations with the genetic polymorphisms is presented in Table S3.

### 3.3. Association between Phenotypic (Measured) and Genotypic Taste Perception and Sleep Patterns

A higher perception of the bitter taste was associated with an earlier waketime and an earlier timing of the midpoint of sleep (*p* < 0.05) (Table 6). Our results also indicated that individuals with a greater score for the salty (*p* = 0.009) and sour (*p* = 0.002) taste perception tests had a higher social jetlag. No significant associations were detected between sleep patterns and sweet perception or CLOCK-rs4580704. Table S1 shows the associations between the measured taste perception variables and the significant sleep patterns separated by the weekdays and weekend.

We replicated the bitter phenotypic results shown above in the selected candidate genes (Table 7). Being a carrier of the taster allele (CC) in TAS2R38-rs713598 was similarly associated with an earlier wake timing (*p* = 0.024) and an earlier midpoint of sleep (*p* = 0.019). FTO-rs9939609 was associated with the waketime (*p* = 0.039), midpoint of sleep (*p* = 0.008), and social jetlag (*p* = 0.028), but this last association was lost when we adjusted for additional confounders (*p* = 0.141). Thus, the obesity risk allele (AA) was related to a later waketime and a later midpoint of sleep. In the additional multivariate-adjusted model adjusted for sex, age, obesity, smoking, and medication, we found the same statistically significant differences, except for in TAS2R38-rs713598 and wake timing, where we obtained a trend (*p* = 0.091). Table S2 shows the associations between the analyzed polymorphisms and the significant sleep patterns of the weekdays and weekend.

### 3.4. Association between Candidate Genes and Obesity-Related Phenotypes: Selected Gene–Pattern Interactions

Finally, to determine the role of the modulating effect of eating and sleep patterns in the association between the polymorphisms and obesity parameters, we carried out a gene–pattern interactions analysis. The three polymorphisms evaluated were associated with obesity after adjustments for sex, age, smoking, medication, and low physical activity. The tasters of the bitter taste, or the carriers of the CC genotype in TAS2R38-rs713598, had a higher BMI (β = 0.87 ± 0.37; *p* = 0.020) and waist circumference (β = 2.55 ± 0.93; *p* = 0.007). FTO-rs9939609 was related to the BMI (β = 0.63 ± 0.34; *p* = 0.022), and the carriers of the AA genotype presented higher values of these parameters. On the other hand, the carrier of the wild allele (G) in rs4580704 had a lower BMI (β = −1.10 ± 0.36; *p* = 0.003) and waist circumference (β = −2.41 ± 0.90; *p* = 0.008). The mean values for each genotype are shown in Table S4. 

**Figure 1:**
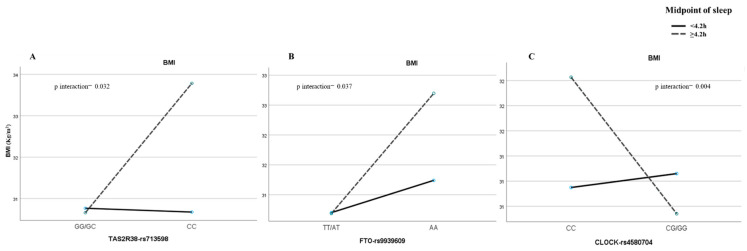
Polymorphism interactions with midpoint of sleep. (**A**) BMI values according to midpoint of sleep by genotypes in TAS2R38-rs71359. (**B**) BMI values according to midpoint of sleep separated by G-allele carriers and AA genotype in FTO-rs9939609. (**C**) BMI values according to midpoint of sleep by genotypes in CLOCK-rs4580704. BMI: body mass index.

We also studied the role of the interactions between sleep and eating patterns and the candidate genes in determining the parameters associated with obesity. We identified statistically significant interactions between TAS2R38-rs713598 and the social jetlag regarding the BMI (*p* = 0.032) and waist circumference (*p* = 0.015). The same gene–pattern interactions were found for TAS2R38-rs713598 and the midpoint of sleep in the same obesity parameters, the BMI (*p* = 0.032) and waist circumference (*p* = 0.010). Thus, only for the CC genotype, a later midpoint of sleep was related to a higher BMI (β = 2.63 ± 1.29; *p* = 0.051), but these differences were not detected for G carriers (β = −0.40 ± 0.48; *p* = 0.934) (Figure 1A).

Due to the novelty and relevance of this gene–sleep pattern interaction, we tested its association with the other candidate genes. We also noticed a midpoint of sleep and FTO-rs9939609 interaction with the BMI (*p* = 0.037). The BMI was higher when the midpoint of sleep was later only for the AA genotype (AA: β = 2.08 ± 1.15; *p* = 0.079) (TT/AT: β = −0.25 ± 0.49; *p* = 0.607) (Figure 1B). Despite not finding any statistically significant associations between rs4580704 in the CLOCK gene and eating and sleep patterns, we found some interesting gene–sleep pattern interactions. Just as we detected for the other SNPs, the sleep midpoint modulated the associations between this polymorphism and the obesity parameters. Thus, the midpoint of sleep also interacted with CLOCK-rs4580704 regarding the BMI (*p* = 0.004) and waist circumference (*p* = 0.028). Later, the midpoint of sleep was positively associated with the BMI in the CC genotype (β = 1.91 ± 0.71; *p* = 0.009) and was negatively associated with the G carriers (β = −1.19 ± 0.59; *p* = 0.048) (Figure 1C). Regarding time patterns, we only found statistically significant interactions with rs4580704. Therefore, a gene–waketime interaction was found regarding the BMI (*p* = 0.006) with a later waketime being related to a higher BMI only in the CC genotype (β = 0.96 ± 0.42; *p* = 0.022) and not in the G carriers (β = −0.69 ± 0.39; *p* = 0.080).

## 4. Discussion

In this study, we characterized the late sleep time and mealtime pattern in the Spanish Mediterranean population. As far as we know, only two studies have explicitly described the timing of both lifestyle factors in the population of this area, both of those obtaining similar times to those shown in our findings (breakfast at 8:00; lunch at 14:00, and dinner at 21:00) [51,52]. However, the waketime in our population was earlier than in these studies, probably due to a greater variability of timeframes as our sample size was larger. Others, in line with our results, have shown that Mediterranean populations have later mealtimes than other regions of Europe [53] or the United States [22]. The results from the EPIC study showed pronounced differences in the mealtimes across Europe, with later meal timetables but a higher load during the day in Mediterranean countries [53]. In agreement with this statement, we observed an association between greater adherence to the MedDiet and afternoon teatime, a meal that takes place after 18:00 in our population. No other associations between the Mediterranean eating pattern and other eating patterns were observed. On the other hand, we found an association between MedDiet adherence and a longer sleep duration. Our group showed lower odds of a short sleep duration in those with a higher adherence to the MedDiet in the Multi-Ethnic Study of Atherosclerosis (MESA) [54]. Also, in a recent review, we remarked that the MedDiet dietary pattern is the one that is the most consistently associated with good quality sleep, mostly due to the high consumption of nuts, vegetables, fruits, legumes, and whole grains, notable sources of tryptophan and melatonin [55]. 

We provided novel evidence for the association between taste perception and eating patterns in the general Mediterranean population. We found an inverse association between the intensity of measured bitter taste perception, the eating midpoint, and breakfast timing. Moreover, these results were consistent with bitter taste perception measured by using a genetic polymorphism (TAS2R38-rs713598) as an instrumental variable in a Mendelian randomization approach. This genetic association increased the causality level of the association between bitter taste perception and eating patterns, as the genetic taste measure is a long-term characteristic versus the measured taste perception that can have a high variability and more dependence on the environmental variables. The ability to perceive a bitter taste is an inherited trait that has been the subject of study for decades [56]. Many cruciferous vegetables and other foods contain this taste [57], so it can influence preferences for dietary choices and can, consequently, affect health [56,58]. To our surprise, taste perception was not associated with adherence to the MedDiet despite other authors having found differences in food patterns in different tastant profiles in the elderly Mediterranean population [59]. Our results could not provide definitive evidence with the present data, as we were the first to analyze eating patterns measured with mealtimes and taste perception. However, the current literature shows results related to other eating behaviors, such as food intake, with similarly inconclusive results [59,60,61,62]. Additional research through longitudinal and experimental studies is needed to increase the causality level and to investigate the complex mechanisms behind these associations. The presence of earlier mealtimes for bitter tasters and for nontasters of sweet, sour, and umami tastes may be associated with more favorable benefits to health [53]. Eating late in the day has been associated with a higher cardiometabolic risk [19,51,63,64,65], including diabetes and obesity [19,63,65]. In addition, in our work, taste perception was linked to sleep patterns. Lv et al. found a positive association between sleepiness and umami and sour taste intensities [46], and Martelli et al. revealed a direct relationship between sleep duration and sweet perception [43]. However, others detected an impairment of taste perception when they presented a short sleep duration, insomnia, and sleepiness [47]. In relation to food preferences, a short sleep duration has been linked in several studies to a higher preference for sweet foods [44,45,46,66]. As ours was a cross-sectional study, we do not know the direction of this association. However, our results suggested that the sleep pattern could modify food intake by altering the capacity for food intensity perception. 

A study carried out in Murcia (Spain) showed that heritability is an additional important factor for eating patterns in addition to cultural, social, and personal factors. A higher heritability was shown for the midpoint of intake (64%), and a higher heritability was shown for having meals earlier in the day, such as breakfast (56%) or lunch (38%), rather than later in the day, such as dinner, where no significant association was found; these findings confirmed the previous results [67]. In our work, we detected that TAS2R38-rs713598 and FTO-rs9939609, but not CLOCK-rs4580704, were associated with meal timing. Specifically, the risk alleles for both genes presented an earlier meal timing. In relation to the FTO polymorphism, rs9939609 has been related to dietary intake [35], but as far as we know, no evidence exists for relationships with eating timing patterns. Consistent with our results for CLOCK-rs4580704 and eating and sleep patterns, Molina-Montes et al. [3] failed to reach statistical significance between the 12 SNPs in the 6 core clock genes (PER1, PER2, PER3, CRY1, NR1D1, and CLOCK) and the chronobiology variables in the 3183 subjects from the EPIC study. However, a study performed in a laboratory setting found that the internal circadian system regulates the temporality of hunger and appetite [68]. Further exploratory analyses in this field are needed.

Later sleep timing has been associated with a higher risk for cardiometabolic factors in many studies [69,70,71]. However, in a rural Chinese cohort, both late and early midpoints of sleep were associated with a higher prevalence of diabetes [72], probably due to a mismatch between internal circadian rhythms and work schedule obligations in early sleepers [73]. We have shown that later sleep timing characteristics are associated with taste perception, specifically a lower bitter taste perception and TAS2R38-rs713598, and with a higher taste intensity for salty and sour tastes. We believe that this relationship with taste perception could partly explain the association found between later sleep times and a poorer diet-quality consumption [74,75] as Garcia-Bailo proposed in a review focused on taste genetic variation and food choices [76]. Moreover, the risk allele in FTO-rs9939609 was related to a later waketime and midpoint of sleep in our study. To date, no study has analyzed the relationship between this polymorphism and sleep patterns. However, there is evidence that AA carriers have a poor diet quality [77,78], and a poor diet quality has been related to a poor sleep quality [55].

Lastly, previous studies have found differences in the obesity risk related to TAS2R38-rs713598 [79,80], FTO-rs9939606 [81,82,83], and CLOCK-rs4580704 [3,8,84], which is consistent with our findings. In our work on sleep and eating patterns, we found a modulating effect only on the midpoint of sleep for all three genes. The midpoint of sleep was correlated with the dim light melatonin onset (DLMO), a marker of the internal circadian phase and the onset of the biological night [72,85,86]. Eating closer or after DLMO was related to higher body fat [86] and glucose levels [87], possibly due to a prolonged glucose spike during the night [85]. In our population, gene–lifestyle interactions were detected for the BMI and waist circumference, with a higher obesity component observed only when the risk genotype for the candidate genes presented a later midpoint of sleep. As far as we know, only two studies have analyzed the interactions between sleep characteristics and genes [48,88]. In a cohort of 297 children aged between 5 and 9 y, an FTO-rs9939609-sleep-duration interaction was noticed. A reduction in the total sleep duration was associated with a worse metabolic profile (BMI, waist circumference, visceral fat, systolic blood pressure, and HOMA-IR) in TT homozygotes but not in AA genotype for these polymorphisms; this relationship was in the opposite direction of those found in our general adult population. In agreement with our findings, Celis-Morales found significant interactions between a polygenic risk score (PRS) of obesity, evaluated with the BMI and waist circumference, and some sleep characteristics. The impact of the PRS on the obesity parameters was stronger when participants from the UK Biobank slept less than 7 or more than 9 h per night, were shift workers, or reported being an evening chronotype [48]. These results highlighted the need for adopting a healthy lifestyle, specifically adequate sleep, which can moderate or intensify the genetic predisposition to obesity in some individuals.

Our study had some limitations and strengths. Our sample size was relatively small, and it was a cross-sectional study; thus, longitudinal and experimental studies are needed to increase the causality level. However, in addition to the measured taste perception phenotypes that could be biased by the cross-sectional assessment, we analyzed genetic polymorphisms as instrumental variables. Thus, for bitter taste perception, we obtained similar associations when TAS2R38-rs713598 was used as a genetic proxy in a Mendelian randomization approach, increasing the level of causality in this study. As sleep was not the primary goal in this cohort, we did not have information about sleep disorders in this sample, which is a possible limiting factor for the results. Otherwise, these results may not be generalizable to other populations since these eating and sleep patterns may be very typical of this area by the Mediterranean Sea. Therefore, more studies are needed in this and other populations for replication and further experimental characterization.

## 5. Conclusions

Our results confirmed the late timing of meals in the Spanish Mediterranean population. Moreover, a greater adherence to the Mediterranean diet pattern was not associated with the times of the main meals or their variability, but it was associated with a longer sleep duration. We also showed, for the first time, an association between a greater bitter taste perception and earlier meal and sleep times. However, the ability to perceive tastes was not associated with adherence to the MedDiet pattern in this sample, apart from an association with sweet taste perception. Lastly, novel gene–environment interactions were detected when examining the obesity-related phenotypes, with participants with a later midpoint being those who presented higher values of the obesity phenotypes analyzed, mainly for the risk alleles in the candidate genes. Such interactions may have a future application in precision health in terms of encouraging susceptible individuals to consider sleep patterns that promote a healthy lifestyle rather than those that may have led to the current high prevalence of obesity. Finally, additional studies are needed to confirm these findings in the Mediterranean population and to extend them to other populations with different cultural patterns.

## Figures and Tables

**Table 1:** Descriptive characteristics of the studied sample and those per sex.

	Total (*n* = 412)	Male (*n* = 141)	Female (*n* = 271)	*p*
Age (years)	46.5 ± 13.9	45.6 ± 14.8	47.0 ± 13.4	0.333
BMI (kg/m^2^)	27.9 ± 5.2	29.0 ± 4.9	27.3 ± 5.3	0.002
Waist circumference (cm)	92.5 ± 15.1	101.0 ± 15.1	88.2 ± 13.2	<0.001
SBP (mm Hg)	125.6 ± 17.2	131.1 ± 15.5	122.1 ± 17.1	<0.001
DBP (mm Hg)	78.8 ± 10.1	82.0 ± 11.2	77.1 ± 9.0	<0.001
Total cholesterol (mg/dL)	213.6 ± 41.0	207.8 ± 40.3	216.7 ± 40.1	0.036
LDL cholesterol (mg/dL)	139.5 ± 32.6	139.9 ± 32.6	139.3 ± 32.6	0.857
HDL cholesterol (mg/dL)	59.7 ± 14.2	51.2 ± 10.3	64.2 ± 13.9	<0.001
Triglycerides (mg/dL)	112.4 ± 79.6	127.8 ± 91.9	104.4 ± 71.3	0.004
Fasting glucose (mg/dL)	95.0 ± 19.5	98.2 ± 23.4	93.4 ± 17.0	0.017
Adherence to MedDiet	8.5 ± 2.2	8.5 ± 2.0	8.5 ± 2.2	0.869
Obesity cases (%)	30.9	37.1	27.8	0.038
Type 2 diabetes (%)	4.5	5.8	3.7	0.235
Current smokers (%)	19.0	15.7	20.7	0.142
Low physical activity (%)	32	25.6	35.2	0.032
Bitter (PTC)	2.0 ± 1.6	1.6 ± 1.5	2.2 ± 1.6	<0.001
Sweet	1.9 ± 1.1	1.7 ± 0.9	2.0 ± 1.2	0.005
Salty	2.6 ± 1.3	2.3 ± 1.3	2.7 ± 1.3	0.009
Sour	2.7 ± 1.3	2.4 ± 1.3	2.9 ± 1.4	<0.001
Umami	2.0 ± 1.4	1.9 ± 1.3	2.0 ± 1.4	0.744

Values are presented as mean ± standard deviation (SD) for continuous variables and % for categorical variables. BMI indicates body mass index; SBP indicates systolic blood pressure, DBP indicates diastolic blood pressure; MedDiet indicates Mediterranean Diet; *p* indicates *p*-values for the comparisons (means or %) between men and women; obesity is indicated by BMI ≥ 30 kg/m^2^; and type 2 diabetes is indicated by antidiabetic drug use or glucose ≥ 126 mg/dL.

**Table 2:** Descriptive characteristics of sleep patterns in the whole sample and those per sex.

	Total (*n* = 412)	Male (*n* = 141)	Female (*n* = 271)	*p*
Sleep duration (h)	7.8 ± 0.9	7.7 ± 1.0	7.8 ± 0.8	0.542
Waketime (hour:min)	7:10 ± 0:53	7:05 ± 0:54	7:13 ± 0:52	0.255
Sleep midpoint (hour:min)	4:13 ± 0:45	04:18 ± 0:54	04:10 ± 0:40	0.233
Social jetlag	1.4 ± 1.0	1.3 ± 1.0	1.4 ± 1.0	0.427

Values are presented as mean ± SD. Sleep timing is represented as hours:minute. Social jetlag is represented as absolute value of integers.

**Table 3:** Descriptive characteristics of the eating patterns in the whole sample and those per sex.

	Total (*n* = 302)	Male (*n* = 110)	Female (*n* = 192)	*p*
Eating window (h)	13.3 ± 1.0	13.3 ± 1.0	13.3 ± 1.0	0.785
Eating midpoint (hour:min)	14:53 ± 0:34	14:54 ± 0:37	14:50 ± 0:32	0.114
Eating jetlag	1.0 ± 0.7	1.0 ± 0.8	1.0 ± 0.6	0.830
Breakfast time (hour:min)	8:10 ± 0:50	8:06 ± 0:49	8:11 ± 0:50	0.488
Mid-morning snack (hour:min)	10:53 ± 0:33	10:38 ± 0:35	11:01 ± 0:29	<0.001
Lunch time (hour:min)	14:39 ± 0:35	14:34 ± 0:32	14:42 ± 0:36	0.041
Evening tea time (hour:min)	18:07 ± 0:35	18:10 ± 0:36	18:05 ± 0:5	0.464
Dinner time (hour:min)	21:31 ± 0:34	21:35 ± 0:35	21:28 ± 0:35	0.080

Values are presented as mean ± SD. Sleep timing is represented as hours:minute. Social jetlag is represented as absolute value of integers.

**Table 4:** Associations between measured taste perception and eating patterns.

Taste (Predictor)	Eating Pattern (Outcome)	β ± SE	*p* ^1^	*p* ^2^
Bitter (PTC)	Eating window	0.03 ± 0.04	0.380	0.476
	Eating midpoint	−0.05 ± 0.02	0.009	0.001
	Eating jetlag	0.05 ± 0.03	0.042	0.501
	Breakfast time	−0.06 ± 0.03	0.057	0.043
	Mid-morning snack	0.05 ± 0.03	0.126	0.678
	Lunch time	−0.03 ± 0.02	0.141	0.105
	Afternoon teatime	−0.02 ± 0.03	0.577	0.523
	Dinner time	−0.04 ± 0.02	0.061	0.009
	Adherence to MedDiet	−0.08 ± 0.07	0.058	0.249
Sweet	Eating window	−0.08 ± 0.05	0.140	0.081
	Eating midpoint	0.08 ± 0.03	0.011	0.030
	Eating jetlag	0.13 ± 0.04	<0.001	0.031
	Breakfast time	0.14 ± 0.05	0.002	0.002
	Mid-morning snack	0.08 ± 0.05	0.092	0.610
	Lunch time	0.01 ± 0.03	0.661	0.711
	Afternoon teatime	0.08 ± 0.04	0.071	0.100
	Dinner time	0.04 ± 0.03	0.190	0.430
	Adherence to MedDiet	0.23 ± 0.10	0.106	0.020
Salty	Eating window	−0.02 ± 0.04	0.567	0.477
	Eating midpoint	0.04 ± 0.02	0.087	0.147
	Eating jetlag	0.07 ± 0.03	0.024	0.178
	Breakfast time	0.07 ± 0.04	0.063	0.063
	Mid-morning snack	0.01 ± 0.04	0.723	0.337
	Lunch time	0.03 ± 0.03	0.267	0.315
	Afternoon teatime	0.06 ± 0.04	0.128	0.148
	Dinner time	0.03 ± 0.02	0.308	0.469
	Adherence to MedDiet	0.10 ± 0.08	0.433	0.233
Sour	Eating window	−0.01 ± 0.04	0.786	0.630
	Eating midpoint	0.04 ± 0.03	0.086	0.207
	Eating jetlag	0.09 ± 0.03	0.005	0.164
	Breakfast time	0.07 ± 0.04	0.048	0.048
	Mid-morning snack	0.05 ± 0.04	0.168	0.928
	Lunch time	0.04 ± 0.03	0.080	0.090
	Afternoon teatime	0.02 ± 0.04	0.562	0.672
	Dinner time	0.03 ± 0.03	0.257	0.552
	Adherence to MedDiet	0.15 ± 0.08	0.267	0.067
Umami	Eating window	−0.03 ± 0.04	0.502	0.365
	Eating midpoint	0.05 ± 0.02	0.030	0.127
	Eating jetlag	0.03 ± 0.03	0.270	0.539
	Breakfast time	0.08 ± 0.04	0.036	0.031
	Mid-morning snack	0.08 ± 0.04	0.037	0.261
	Lunch time	−0.01 ± 0.02	0.654	0.759
	Afternoon teatime	0.02 ± 0.04	0.654	0.832
	Dinner time	0.03 ± 0.02	0.221	0.656
	Adherence to MedDiet	0.11 ± 0.08	0.464	0.159

Results of multivariable linear regressions. *p* ^1^ represents unadjusted *p* values, *p* ^2^ represents *p* values adjusted for sex and age (additive model), PTC represents phenylthiocarbamide, and MedDiet represents Mediterranean diet.

**Table 5:** Associations between selected polymorphisms and eating patterns.

Gene (Predictor)	Eating Pattern (Outcome)	β ± SE	*p* ^1^	*p* ^2^
TAS2R38-rs713598	Eating window	0.13 ± 0.09	0.155	0.163
	Eating midpoint	−0.13 ± 0.05	0.011	0.008
	Eating jetlag	0.00 ± 0.06	0.988	0.831
	Breakfast time	−0.17 ± 0.08	0.029	0.029
	Mid-morning snack	0.12 ± 0.08	0.126	0.265
	Lunch time	−0.07 ± 0.05	0.204	0.205
	Afternoon teatime	0.05 ± 0.08	0.554	0.495
	Dinner time	−0.06 ± 0.05	0.277	0.218
	Adherence to MedDiet	−0.09 ± 0.17	0.580	0.707
FTO-rs9939609	Eating window	−0.14 ± 0.08	0.076	0.063
	Eating midpoint	0.04 ± 0.05	0.442	0.669
	Eating jetlag	−0.1 ± 0.06	0.813	0.365
	Breakfast time	0.09 ± 0.07	0.210	0.197
	Mid-morning snack	0.11 ± 0.06	0.084	0.336
	Lunch time	−0.11 ± 0.05	0.014	0.021
	Afternoon teatime	−0.13 ± 0.07	0.046	0.038
	Dinner time	−0.05 ± 0.05	0.319	0.146
	Adherence to MedDiet	−0.16 ± 0.15	0.300	0.471
CLOCK-rs4580704	Eating window	0.04 ± 0.08	0.627	0.684
	Eating midpoint	−0.03 ± 0.05	0.491	0.281
	Eating jetlag	0.07 ± 0.06	0.254	0.459
	Breakfast time	−0.02 ± 0.07	0.759	0.787
	Mid-morning snack	−0.05 ± 0.08	0.519	0.536
	Lunch time	0.02 ± 0.05	0.653	0.522
	Afternoon teatime	−0.07 ± 0.07	0.332	0.268
	Dinner time	0.00 ± 0.05	0.928	0.747
	Adherence to MedDiet	0.09 ± 0.17	0.576	0.435

Results of multivariable linear regressions. *p* ^1^ represents unadjusted *p* values, *p* ^2^ represents *p* values adjusted for sex and age (additive model), and MedDiet represents Mediterranean Diet.

**Table 6:** Associations between phenotypic taste perception and sleep patterns.

Taste (Predictor)	Sleep Pattern (Outcome)	β ± SE	*p* ^1^	*p* ^2^
Bitter (PTC)	Waketime	−0.06 ± 0.03	0.020	<0.001
	Total Sleep Duration	0.02 ± 0.03	0.594	0.875
	Midpoint of sleep	−0.06 ± 0.03	0.034	0.009
	Social jetlag	0.06 ± 0.04	0.110	0.766
Sweet	Waketime	0.06 ± 0.04	0.128	0.465
	Total Sleep Duration	−0.01 ± 0.05	0.811	0.426
	Midpoint of sleep	0.01 ± 0.04	0.894	0.990
	Social jetlag	0.10 ± 0.06	0.070	0.341
Salty	Waketime	−0.01 ± 0.03	0.850	0.425
	Total Sleep Duration	−0.05 ± 0.04	0.256	0.157
	Midpoint of sleep	−0.02 ± 0.04	0.642	0.635
	Social jetlag	0.13 ± 0.04	0.004	0.009
Sour	Waketime	0.03 ± 0.03	0.374	0.984
	Total Sleep Duration	0.00 ± 0.04	0.994	0.586
	Midpoint of sleep	0.02 ± 0.04	0.483	0.617
	Social jetlag	0.18 ± 0.04	<0.001	0.002
Umami	Waketime	0.01 ± 0.03	0.780	0.575
	Total Sleep Duration	−0.05 ± 0.04	0.254	0.127
	Midpoint of sleep	0.04 ± 0.04	0.252	0.393
	Social jetlag	0.09 ± 0.05	0.051	0.258

Results of multivariable linear regressions. *p* ^1^ represents unadjusted *p* values, *p* ^2^ represents *p* values adjusted for sex and age (additive model), and PTC represents phenylthiocarbamide.

**Table 7:** Associations between selected polymorphisms and sleep patterns.

Gene (Predictor)	Sleep Pattern (Outcome)	β ± SE	*p* ^1^	*p* ^2^
TAS2R38-rs713598	Waketime	−0.14 ± 0.07	0.036	0.024
	Total Sleep Duration	0.01 ± 0.08	0.933	0.783
	Midpoint of sleep	−0.14 ± 0.09	0.118	0.019
	Social jetlag	0.02 ± 0.09	0.865	0.592
FTO-rs9939609	Waketime	0.15 ± 0.06	0.015	0.039
	Total Sleep Duration	0.08 ± 0.07	0.304	0.295
	Midpoint of sleep	0.18 ± 0.06	0.005	0.008
	Social jetlag	−0.15 ± 0.08	0.061	0.028
CLOCK-rs4580704	Waketime	0.03 ± 0.07	0.603	0.787
	Total Sleep Duration	−0.07 ± 0.08	0.433	0.398
	Midpoint of sleep	0.11 ± 0.07	0.147	0.194
	Social jetlag	0.07 ± 0.09	0.425	0.570

Results of multivariable linear regressions. *p* ^1^ represents unadjusted *p* values; *p* ^2^ represents *p* values adjusted for sex and age (additive model).

## Data Availability

Neither the participants’ consent forms nor the ethics approval included permission for open access. However, we followed a controlled data-sharing collaboration model, and data for collaborations will be available upon request pending application and approval. Investigators who are interested in this study can contact the corresponding author, Dolores Corella (dolores.corella@uv.es).

## Supplementary Materials

The following supporting information can be downloaded at https://www.mdpi.com/article/10.3390/nu15030708/s1, Table S1: Association between measured taste perception and eating/sleep pattern taking into account the days of the week (weekdays or weekend); Figure S1: Association between adherence to Mediterranean diet and total sleep duration; Table S2: Prevalence of the genotypes of the SNPs studied; Table S3: Association between the selected polymorphisms and eating/sleep pattern taking into account the days of the week (weekdays or weekend); Table S4: Means, standard deviations, and *p*-values of body mass index (BMI) and waist circumference according to the indicated genotypes.
