# Supplementary material for: Timing of Meals and Sleep in the Mediterranean Population: The Effect of Taste, Genetics, Environmental Determinants, and Interactions on Obesity Phenotypes
Source: Nutrients. 2023 Jan 30;15(3):708. doi: 10.3390/nu15030708 (PMC9921798; doi:10.3390/nu15030708)
Supplement: Supplementary file 1 [file nutrients-15-00708-s001.zip › nutrients-2145310-supplementary.pdf]

SUPPLEMENTARY MATERIAL

Timing of meals and sleep in a Mediterranean population: Taste, genetics, environmental determinants and interactions on obesity phenotypes

Barragán R, Fernández-Carrión R, Asensio-Márquez E.M, Ortega-Azorín C, Alvarez-Sala A, Pérez-Fidalgo A, Sorlí J.V, Portolés O, Gonzalez-Monje I, St-Onge M-P, Corella D

Table S1: Association between the measured taste perception and eating/sleeping pattern taking into account the days of the week (weekdays or weekend)

Taste	Eating/Sleeping pattern	β±SE	P[1]
Bitter (PTC)	Eating midpoint weekdays	-0.07 ± 0.02	0.001
	Eating midpoint weekend	-0.05 ± 0.03	0.042
	Breakfast time weekdays	-0.08 ± 0.04	0.021
	Breakfast time weekend	-0.04 ± 0.04	0.338
	Dinner time weekdays	0.06 ± 0.02	0.013
	Dinner time weekend	-0.04 ± 0.02	0.046
	Wake time weekdays	-0.09 ± 0.03	0.005
	Wake time weekend	-0.10 ± 0.04	0.005
	Midpoint of sleep weekdays	-0.10 ± 0.03	0.001
	Midpoint of sleep weekend	-0.08 ± 0.04	0.043
Sweet	Eating midpoint weekdays	0.04 ± 0.03	0.190
	Eating midpoint weekend	0.12 ± 0.04	0.002
	Breakfast time weekdays	0.09 ± 0.05	0.098
	Breakfast time weekend	0.20 ± 0.05	<0.001
Sour	Breakfast time weekdays	0.03 ± 0.04	0.444
	Breakfast time weekend	0.08 ± 0.04	0.066
Umami	Breakfast time weekdays	0.06 ± 0.04	0.139
	Breakfast time weekend	0.05 ± 0.04	0.224

Results of multivariable linear regressions. P1: Unadjusted P value; P2 Adjusted P value for sex and age (additive model). PTC: phenylthiocarbamide; MedDiet: Mediterranean Diet.

Figure S1. Association between adherence to Mediterranean Diet and total sleep duration.

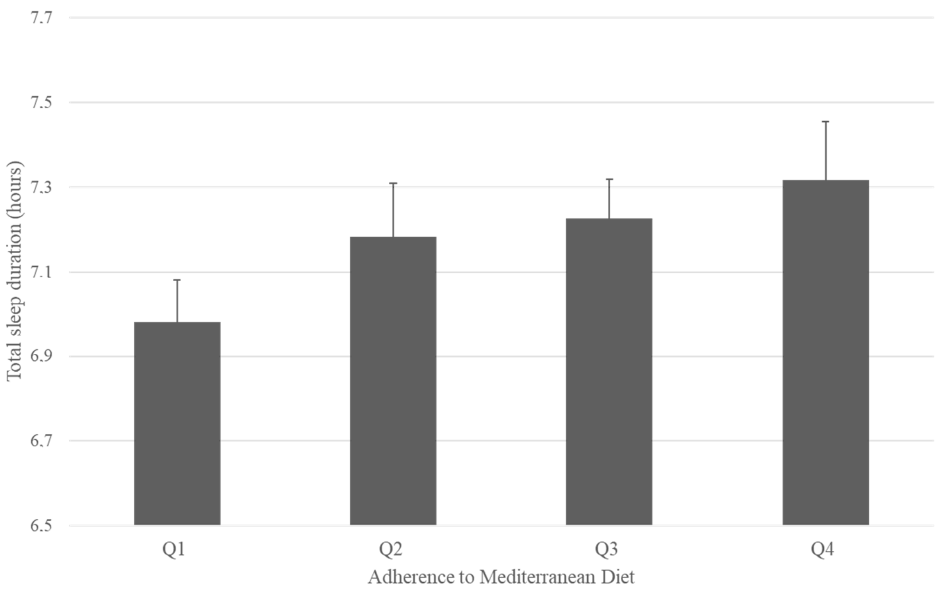

Q: quartile. Q1 was for a score between 3 to 6; Q2 for a score of 8; Q3 for a score of 9-10; and Q4 for a score ≥11 (P=0.010)

Table S2. Prevalence of the genotypes of the SNPs studied.

Gene	SNP	Genotype	Population (%)	Allele frequency
**TAS2R38**	rs713598	GG	29.8	G=0.562<br>C=0.438
		GC	52.8	
		CC	17.4	
**FTO**	rs9939609	TT	37.4	T=0.592<br>A=0.408
		TA	43.7	
		AA	18.9	
**CLOCK**	rs4580704	CC	39.3	C=0618<br>G=0.382
		CG	46.9	
		GG	13.9	

A: adenine; C: cytosine; G: guanine; T: thymine.

Table S3: Association between the selected polymorphism and eating/sleeping pattern taking into account the days of the week (weekdays or weekend)

Gene	Eating/Sleeping pattern	β±SE	P[1]
TAS2R38-rs713598	Eating midpoint weekdays	-0.13 ± 0.05	0.015
	Eating midpoint weekend	-0.13 ± 0.07	0.039
	Breakfast time weekdays	-0.20 ± 0.08	0.020
	Breakfast time weekend	-0.07 ± 0.09	0.472
	Wake time weekdays	-0.16 ± 0.07	0.030
	Wake time weekend	-0.10 ± 0.09	0.225
	Midpoint of sleep weekdays	-0.12 ± 0.07	0.028
	Midpoint of sleep weekend	-0.20 ± 0.08	0.017
FTO-rs9939609	Lunch time weekdays	-0.12 ± 0.06	0.040
	Lunch time weekend	-0.06 ± 0.04	0.163
	Afternoon teatime weekdays	-0.12 ± .0.06	0.056
	Afternoon teatime weekend	-0.04 ± 0.07	0.561
	Wake time weekdays	0.16 ± 0.07	0.020
	Wake time weekend	0.05 ± 0.08	0.510
	Midpoint of sleep weekdays	0.16 ± 0.06	0.012
	Midpoint of sleep weekend	0.06 ± 0.08	0.469

Results of multivariable linear regressions. P1: Unadjusted P value; P2 Adjusted P value for sex and age (additive model). PTC: phenylthiocarbamide; MedDiet: Mediterranean Diet.

Table S4. Means, standard deviations and p-values of body mass index (BMI) and waist circumference according to the indicated genotypes.

Genotype	BMI (kg/m^2)		Waist circumference (cm)	
rs713598				
GG	27.0±5.4	P=0.040	90.9±15.7	P=0.071
GC	28.1±4.8		92.8±13.5	
CC	27.9±5.2		96.3±18.6	
rs9939609				
TT	27.5±5.0	P=0.283	92.3±15.8	P=0.766
TA	28.0±5.1		92.6±14.5	
AA	28.6±5.6		93.9±15.5	
rs4580704				
CC	28.6±5.1	P=0.033	94.7±14.8	P=0.117
CG	27.6±5.4		91.6±15.6	
GG	26.5±4.7		90.8±14.7	

Values are mean ± SE. P values were obtained by ANOVA analysis (unadjusted). BMI: Body Mass Index.
